# Remarks on Filling or Plugging Teeth

**Published:** 1844-11-02

**Authors:** J. F. B. Flagg

**Affiliations:** Surgeon Dentist


### REMARKS ON FILLING OR PLUGGING TEETH.

BY J. F. B. FLAGG, M. D., SURGEON DENTIST.

In offering my opinions in relation to the advantages or disadvantages of this operation, according to the faithfulness with which it may be performed, and the material used therefor, I cannot but entertain a hope that medical men, generally, may be led to feel sufficient interest in the matter, at least, to observe attentively those cases which often present themselves in connection with other diseases submitted to their care.

I would, in the first place, ask attention to some of the articles in common use at the present day, which not only are injurious to the teeth themselves, but in very many cases lead to inflammation of the *periosteum*, entire destruction of that membrane, "*gumboils*, *ulceration*, inflammation extending to the neighbouring teeth, swelling of the glands about the tongue, throat and neck, neuralgia about the jaws, face and temples; and where several large fillings are placed at about the same time, in very hollow teeth, even *salivation* is produced in those who are highly susceptible to the influence of *mercury*." All metallic pastes being composed of hydrargyrum principally, are subjected to the greatest objection as leading to the above mentioned results. There may be a few men honest in their ignorance in the use of this article; that is, sufficiently so to prepare the tooth for its reception in as perfect a manner as they are capable of, by previously removing the decayed substance, and giving the material healthy bone to rest upon;—when this care is taken, the pernicious effects of the mercurial amalgam are more immediately felt; and, consequently the most approved method of using it would appear to be, at once to plaster up the hole, regardless of any previous manipulation. The effect of stopping a tooth under the last mentioned circumstances is too obvious to require any farther illustration.

That cement which most deservedly stands at the head of these vile nostrums is known by the name of "*Royal Succedaneum*," introduced into New York by those *princes* of dental humbugs, the Messrs. Crawceur, about ten years since; after a very successful run of two or three months in that city, they suddenly left for Europe; several who had been duped by them, chartered a steam-boat, and fireing up gave hot pursuit; failing to overtake them, they returned to the city, if with dry eyes, with water enough in their mouths to make up for it. The success of these fellows has given birth to many other inventions of similar type, whereby the public are not only cheated out of their money but out of their teeth, and, in many instances, their health also.

The principal difficulties which present themselves in the use of those cements known by various names, such as "*Enamel Cement*," "*Mineral Paste*," "*Lithodeon*," &c. &c., seem to be, that if sufficient mercury be not added to the material to enable them to convert it into a paste at a temperature but little above that of the body, the cooling process, after placing it within the tooth will cause it to shrink, and thus endanger its security within the cavity;—whereas, on the other hand, mercurial preparations of this character, cause the teeth to grow black, will decompose good and perfect fillings of gold in other parts of the mouth, and generate galvanic action, serious and alarming in all its consequences.

In an article communicated for the Boston Medical and Surgical Journal, by Dr. J. F. Flagg, of that city, he closes with these remarks upon this subject:—

"I am fully aware that these *cements* or *amalgams* have been used in some cases where they seem to be of service; but here still is deception;—for in all such that have come under my observation, (and these are very numerous,) it can be demonstrated, by an examination of them, that great mischief is going on beneath such fillings, and that a different and better treatment might have been adopted."

*Tin* and *lead* are often used for the purpose of arresting decay in teeth; the former of these metals, however, much the more frequent. They are both objectionable, upon the ground that they are extremely liable to oxidation; I have, therefore, adopted the plan in my practice, of their entire exclusion, except in occasional cases of doubt as to success in the filling process; in all such cases I will not suffer it to remain in a tooth to exceed a very few weeks; and on becoming satisfied upon this point, I immediately remove it and substitute gold.

Teeth should never be filled with more than one kind of metal; that is to say, with gold, tin, or lead, in the same mouth, the action of the acids upon them, under these circumstances, becomes quite pernicious, and is even more injurious than if they were filled with either of the baser metals alone.

I have now only to consider one other article in common use by the best dentists of the present day, for the purpose of filling teeth, and that is gold. Gold-foil, as it is denominated, should be free from all alloy; it is prepared by a process of hammering it into sheets, and so annealing it as to render it perfectly malleable. This is considered a nice and difficult process, so to prepare it for the most perfect dental operations; although there are many engaged in this country in the business of gold beating, but few have as yet attained much celebrity in this particular branch.

Filling a tooth *properly* with gold, implies two things: in the first place, *skill* is required to prepare the cavity, and so pack the gold as to render the tooth invulnerable to decay at that point for a long course of years; and secondly, *common honesty* is an important requisite where so costly an article as gold is applied to this purpose. I will illustrate this in a plain and simple manner, leaving your readers to apply it to whichever head they please; to one of the two, if not to both, it most certainly belongs. If not the average, a large minority of teeth, requiring filling will consume what costs the dentist *one dollar*. Now, if I should advertise to "fill teeth with gold for from *fifty* to *seventy-five cents* each," I would merely ask how long would it take me to get rich?

The tooth to be stopped should be thoroughly freed from any decayed portion; it should likewise be entirely wiped dry, and no moisture suffered to get into the cavity while packing the gold; and the entire aperture uniformly filled solid, so much so, that a hard pressure with a sharp instrument will make no impression upon that filling; it should then be burnished and left perfectly smooth.

There are various methods of preparing the gold previous to filling, such as cutting it into ribbons, and packing it in that state; some take those ribbons and twist them lightly between the thumb and finger, into the form of a rope; and others again roll up the gold into balls of various shapes and sizes; good operations can be made by each of these methods. Much credit has been reflected upon the profession by the operations in this department, of the late Dr. Hudson and his able cotemporaries, Drs. Gardette and Koecker, particularly when we take into consideration the great difficulties under which they laboured in obtaining material in every respect suitable for this delicate art.

In conclusion, I am happy in presenting my acknowledgments to a few gentlemen in the profession in this city, who are determined to maintain the dignity of their calling, by an uncompromising warfare against that empiricism which has so largely forced itself into our midst.

